# Stroke and the risk of gastrointestinal disorders: A Mendelian randomization study

**DOI:** 10.3389/fneur.2023.1131250

**Published:** 2023-02-21

**Authors:** Jingru Song, Wenjing Chen, Wei Ye

**Affiliations:** Department of Gastroenterology, Hangzhou Traditional Chinese Medicine (TCM) Hospital Affiliated to Zhejiang Chinese Medical University, Hangzhou, Zhejiang, China

**Keywords:** stroke, gastrointestinal disorders, Mendelian randomization, causality, risk

## Abstract

**Background:**

The issue of whether a stroke is causally related to gastrointestinal disorders was still not satisfactorily understood. Therefore, we investigated if there is a connection between stroke and the most prevalent gastrointestinal disorders, including peptic ulcer disease (PUD), gastroesophageal reflux disease (GERD), irritable bowel syndrome (IBS), and inflammatory bowel disease (IBD).

**Methods:**

We applied two-sample Mendelian randomization to investigate relationships with gastrointestinal disorders. We obtained genome-wide association study (GWAS) summary data of any stroke, ischemic stroke, and its subtypes from the MEGASTROKE consortium. From the International Stroke Genetics Consortium (ISGC) meta-analysis, we acquired GWAS summary information on intracerebral hemorrhage (ICH), including all ICH, deep ICH, and lobar ICH. Several sensitivity studies were performed to identify heterogeneity and pleiotropy, while inverse-variance weighted (IVW) was utilized as the most dominant estimate.

**Results:**

No evidence for an effect of genetic predisposition to ischemic stroke and its subtypes on gastrointestinal disorders were found in IVW. The complications of deep ICH are a higher risk for PUD and GERD. Meanwhile, lobar ICH has a higher risk of complications for PUD.

**Conclusion:**

This study provides proof of the presence of a brain–gut axis. Among the complications of ICH, PUD and GERD were more common and associated with the site of hemorrhage.

## 1. Introduction

Stroke is one of the leading causes of death and disability worldwide (1, 2). Based on neuropathology, there are two main categories of stroke: ischemic stroke (IS) and hemorrhagic stroke. Of the two major types of stroke, IS is the more frequent type (3). There are various subtypes of IS, such as large artery stroke, cardioembolic stroke, and small vessel stroke (4). Hemorrhagic stroke includes subarachnoid hemorrhage (SAH) and intracerebral hemorrhage (ICH). After a stroke, most patients will have varying degrees of motor impairment, cognitive impairment, speech dysphagia, depression, and other sequelae (5). In addition, up to 50% of patients usually experience gastrointestinal sequelae (6). The most common gastrointestinal disorders include PUD, GERD, IBS, and IBD. Among these four diseases, the prevalence of GERD is the highest, up to 18.1–27.8% in North America, followed by IBS and PUD, and the prevalence of IBD is lower. Patients with IBD commonly have abdominal pain, diarrhea, and bloody stools, while IBS has abdominal pain and altered bowel habits. GERD is usually characterized by regurgitation symptoms and heartburn, while PUD symptoms are not specific and abdominal pain is common (7–10). They sometimes have similar symptoms, such as abdominal pain, and the development of these disorders is all related to the brain–gut axis (11–13). Some observational studies have given attention to the relationship between stroke and peptic ulcer disease (PUD) (14) and also stroke and gastroesophageal reflux disease (GERD) (15). The study found that the GERD risk of patients with stroke is about 1.51 times that of patients without stroke (15). However, so far, it is not clear whether there is a causal relationship between the two diseases.

A growing number of observational studies have demonstrated complex interactions between stroke and gastrointestinal disorders (16–18). Furthermore, studies have shown that stroke promotes the destruction of the intestinal barrier and the imbalance of gut microbiota (19, 20). These proved that there is bidirectional communication between the brain and the gut, usually referred to as the brain–gut or gut–brain axis (21). After a stroke, the bidirectional communications between the brain and the gut may relate to the dysfunction of the autonomic nervous system, resulting in gastrointestinal disorders (22, 23). However, the exact mechanism accounting for the brain–gut axis is still widely considered as unsatisfactorily understood.

In systematic reviews and meta-analyses, their causal relationship is unclear or confusing. Mendelian randomization (MR) is a research method using a genetic variation to evaluate the causal relationship between exposures and outcomes based on Mendel's second law. MR overcomes the limitations of observational research by exposing potential causal links and has proved valuable in exploring the causality by using single-nucleotide polymorphisms (SNPs). SNPs are required to be associated with exposures and should not be independently associated with outcomes, except through exposures. Furthermore, SNPs must not be associated with confounders (24, 25). Moreover, we can further explore the outcomes of insufficient data in RCT through large samples in the genome-wide association study (GWAS). To our knowledge, there are relatively few studies on the causal relationship between stroke and gastrointestinal disorders, and gastrointestinal disorders have received less attention than other stroke complications, yet gastrointestinal disorders after stroke may lead to poor prognosis or even death (26). PUD, GERD, irritable bowel syndrome (IBS), and inflammatory bowel disease (IBD) are common diseases of the digestive system (27, 28). Therefore, we are committed to studying the causal effects of stroke and its subtypes and common gastrointestinal disorders by applying two-sample Mendelian randomization.

## 2. Material and methods

**Figure 1:**
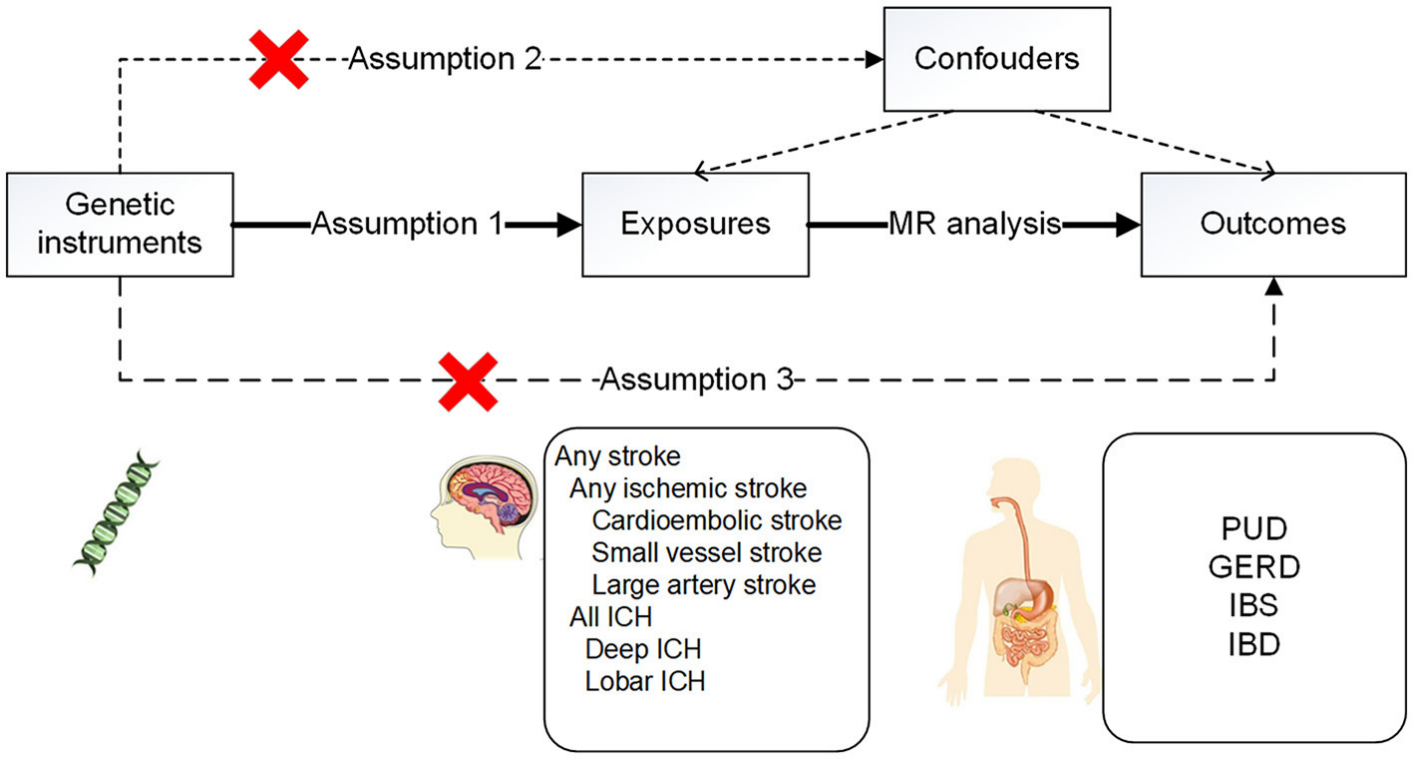
Conceptual MR framework.

The conceptual MR framework is presented in Figure 1.

### 2.1. Study design and ethical approval

According to the Strengthening the Reporting of Observational Studies in Epidemiology-Mendelian Randomization (STROBE-MR) recommendations (29), the MR design was based on three hypotheses: (1) in this investigation, genetic variation was highly linked with the exposure of interest (stroke and its subtypes); (2) genetic variation was not associated with possible confounders; and (3) genetic variation solely had an impact on the outcome through the exposure of interest (gastrointestinal disorders in this study).

### 2.2. Data sources for stroke and gastrointestinal disorders

To investigate the potential causative relationship between stroke and gastrointestinal disorders such as PUD, GERD, IBS, and IBD, we used a two-sample MR method. The largest meta-analysis of genome-wide association studies (GWASs) produced by the MEGASTROKE consortium provided pooled statistics for any stroke, any ischemic stroke, and its subtypes (cardioembolic stroke, small vessel stroke, and large artery stroke) confirmed by clinical and imaging criteria (30). The International Stroke Genetics Consortium (ISGC), a group with European roots, provided the exposure dataset for hemorrhagic stroke (Table 1) (31). Regarding the outcome dataset, we selected the results according to Wu et al. (28). PUD, GERD, IBS, and IBD are common gastrointestinal diseases.

**Table 1:** Details of the GWAS included in the MR.

**Phenotype**	**Data source**	**Sample size**	**%European**
**Exposures**			
Any stroke	MEGASTROKE (30)	40,585 cases/406, 111 controls	100%
Any ischemic stroke	MEGASTROKE (30)	34,217 cases/406,111 control	100%
Cardioembolic stroke	MEGASTROKE (30)	7,193 cases/406,111 control	100%
Small vessel stroke	MEGASTROKE (30)	5,386 cases/406,111 control	100%
Large artery stroke	MEGASTROKE (30)	4,373 cases/406,111 control	100%
All ICH	ISGC (31)	1,545 cases/1,481 controls	100%
Deep ICH	ISGC (31)	664 cases/1,481 controls	100%
Lobar ICH	ISGC (31)	881 cases/1,481 controls	100%
**Outcomes**			
PUD	Wu et al. (28)	16,666 cases/406, 111 controls	100%
GERD	Wu et al. (28)	54,854 cases/401,473 controls	100%
IBS	Wu et al. (28)	29,524 cases/426,803 controls	100%
IBD	Wu et al. (28)	7,045 cases/449,282 controls	100%

ICH, intracerebral hemorrhage; PUD, peptic ulcer disease; GERD, gastroesophageal reflux disease; IBS, irritable bowel syndrome; IBD, inflammatory bowel disease.

### 2.3. Selection of genetic instruments

First, in line with the findings of Kwok et al. (32), we relaxed the correlation threshold with *P* <5 × 10^−6^ and linkage disequilibrium (LD) (*r*^2^ < 0.001) to obtain the top independent SNPs. This was done in light of the small number of SNPs (*P* < 5 × 10^−8^) that reached genome-wide significance. This strategy has been applied extensively in earlier MR investigations (33, 34). Second, the results of MR analysis are believed to be unaffected by weak instrumental bias if there is an F-statistic larger than 10. We used the following:


$$\begin{array}{l} R^{2}=2\times \left(1-MAF\right)\times MAF\times {\left(\beta \right)}^{2},\\ F=\left(\frac{R^{2}}{1-R^{2}}\right)\left(\frac{n-k-1}{k}\;\right). \end{array}$$

Third, we extracted the secondary phenotypes of each SNP from a PhenoScanner V2 (35) and the GWAS library to exclude any putative polymorphism effects. The radial MR and MR pleiotropy residual sum and outlier (MR-PRESSO) tests were used to eliminate outliers before each MR analysis.

### 2.4. Statistical analysis

Three methods, including MR-Egger, weighted median, and random effect inverse-variance weighting (IVW), were utilized in the MR analysis to evaluate robust effects. The primary analysis method was the IVW method with various models, depending on the heterogeneity. At least half of the data for the Mendelian randomization study must originate from reliable instruments to use the weighted median estimator (36, 37). The effectiveness of potential pleiotropic tools must be independent of their direct relationships with the outcome for MR-Egger regression to be valid. Radial MR-Egger was used to estimate the horizontal pleiotropy and to identify outlier variants (38). Heterogeneity was also assessed using Cochran's *Q*-test. With the Cochran Q test (statistics were deemed to be significant if *P* < 0.05) and the intercept from MR-Egger regression (statistics were deemed to be significant if *P* < 0.05), we evaluated heterogeneity between Mendelian randomization estimates. We also evaluated potential directional polymorphisms using funnel plots. We used fixed-effects IVW and limited our instrument selection for sensitivity analyses to a lower LD correlation threshold. In conclusion, we conducted a thorough investigation of causation using all these techniques. Given the 32 MR estimates, the Bonferroni-corrected *P-*value for the study of gastrointestinal disorders was set at 0.05/32 (1.563 × 10^−3^), and *P* < 0.05 was regarded as nominally significant. The statistical study was performed using R (version 4.2.0) and the “TwoSampleMR” and “RadialMR” packages.

## 3. Results

The SNPs of stroke subtypes on gastrointestinal disorders are listed in Supplementary Tables 1–8. Looking over the Phenoscanner, three SNPs (rs10850001, rs10774624, and rs3184504) were associated with smoking and were removed when analyzing PUD-associated SNPs. A total of 10 SNPs (rs12932445, rs1537375, rs2107595, rs2466455, rs4444878, rs4932370, rs6536024, rs6838973, rs72700114, and rs2634074) were related to the anticoagulant use, which was analyzed for PUD-related SNPs removed during the analysis. A total of 10 SNPs (rs10774624, rs1549758, rs1975161, rs2107595, rs2284665, rs34416434, rs42039, rs616154, rs78893982, and rs8103309) were associated with obesity and were removed in the analysis of GERD-related SNPs.

**Figure 2:**
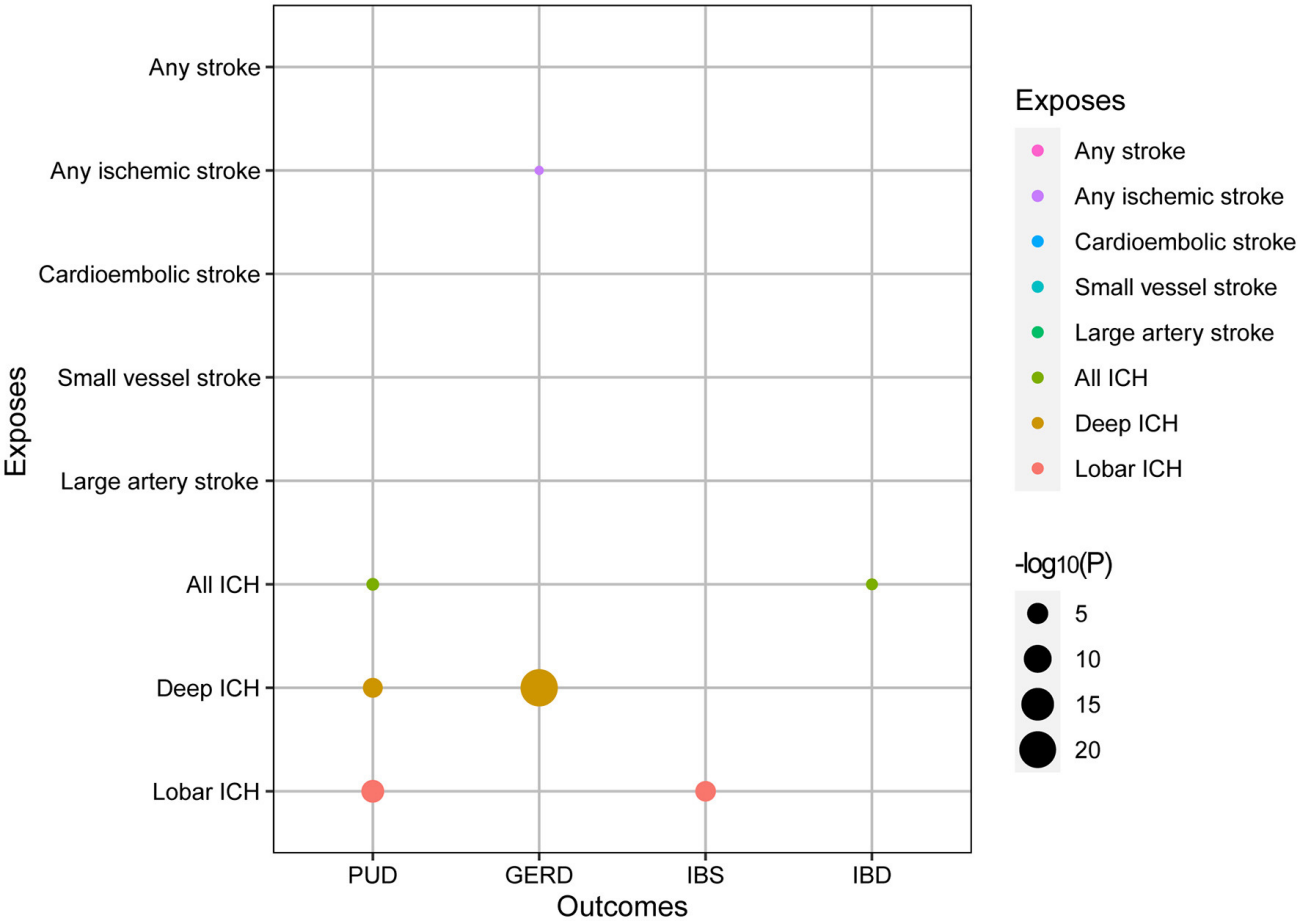
Bubble plot of MR study of stroke on gastrointestinal disorders derived from IVW.

**Figure 3:**
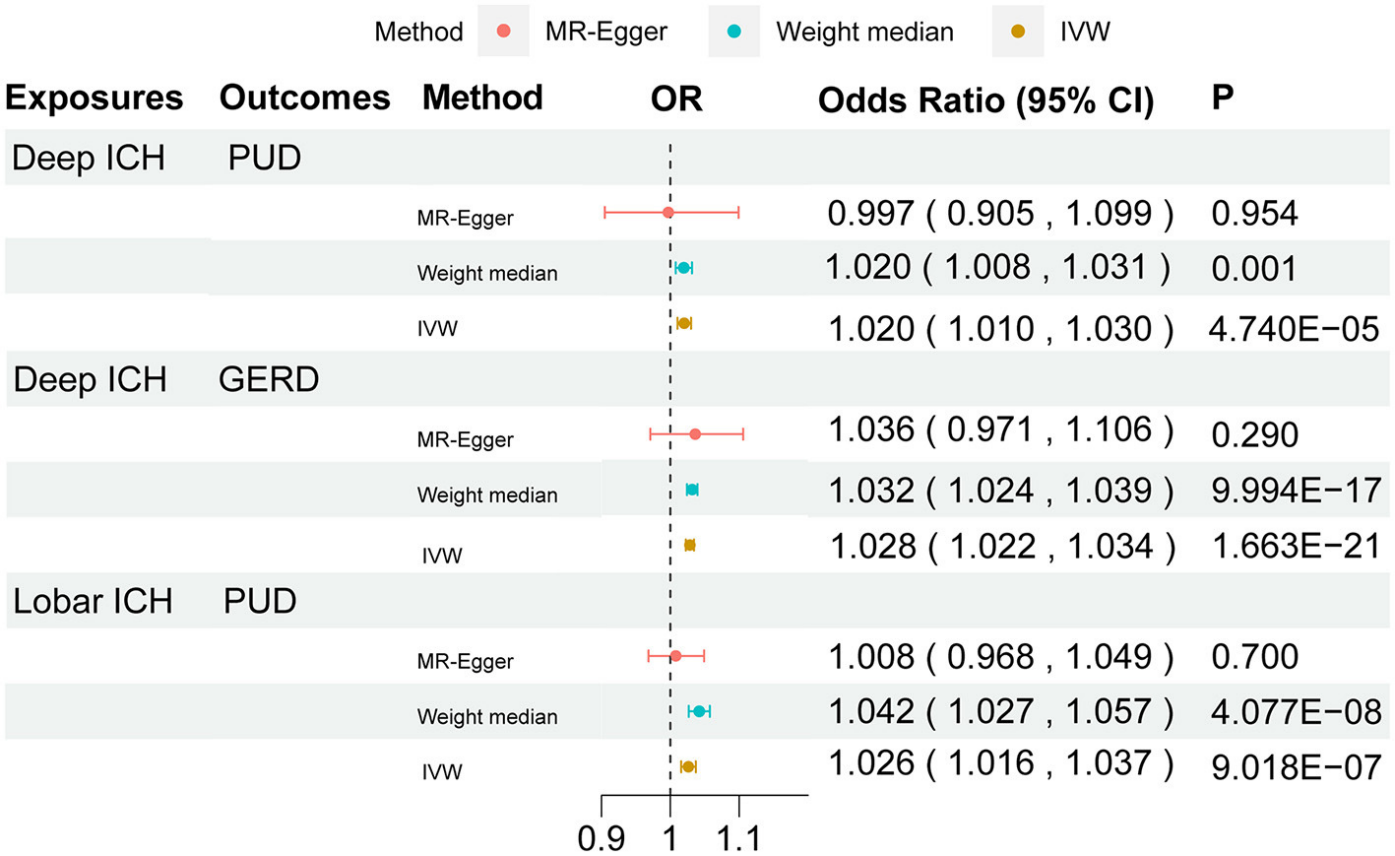
Forest plot for the causal effect of stroke on the risk of gastrointestinal disorders. OR, odds ratio; CI, confidence interval.

We performed a comprehensive MR study of stroke and its subtypes on gastrointestinal diseases (Supplementary Table 9). Among them, using IVW as the primary analysis, it could be seen that genetics predicted that any ischemic stroke had a normal significance with GERD (*P* < 0.05). All ICHs had normal significance with PUD and IBD (*P* < 0.05). Meanwhile, deep ICH had signed with the PUD and GERD (*P* < 1.563 × 10^−3^). Lobar ICH had signed with the PUD and IBS (*P* < 1.563 × 10^−3^). A bubble plot was used to show the statistical significance of the analysis (Figure 2). After that, the MR analyses with significant *P-*values were demonstrated in a forest plot (Figure 3). For ischemic stroke, there was no significant causal relationship with gastrointestinal disorders. For hemorrhagic stroke, the result of IVW showed that deep ICH [odds ratio (OR): 1.020; 95% confidence interval (CI): 1.010–1.030; *P* = 4.740 × 10^−5^] was associated with an increased risk of PUD and greater disease severity with the weight median method (OR: 1.020; 95% CI: 1.010–1.030; *P* = 4.740 × 10^−5^). The results of the MR-Egger method showed consistent directions but were not statistically significant (OR: 0.997; 95% CI: 0.905–1.099; *P* = 0.954). In addition, similar causal estimates of lobar ICH on PUD were obtained, and IVW (OR: 1.026; 95% CI: 1.016–1.037; *P* = 9.018 × 10^−7^) and weight median (OR: 1.042; 95% CI: 1.027–1.057; *P* = 4.077 × 10^−8^) were included, while the same result was observed using the MR-Egger method but without any statistical difference (OR: 1.008, 95% CI: 0.968–1.049, *P* = 0.700). Deep ICH was associated with an increased risk of GERD with the IVW (OR: 1.028; CI: 1.022–1.034; *P* = 1.663 × 10^−21^) and weight median (OR: 1.032; CI: 1.024–1.030; *P* = 9.994 × 10^−17^); however, there was no statistical difference in the MR-Egger method (OR: 1.036; CI: 0.971–1.106; *P* = 0.290), where all *p* > 0.05 for the MR-Egger intercept test, except for the MR analysis of lobar ICH on the IBS of lingual without weight median, indicated no horizontal pleiotropy. For significance and nominal significance estimates, Cochran's *Q*-test, the MR-Egger intercept test, the leave-one-out analysis, and the funnel plot were used to assess horizontal multiplicity (Supplementary Figures 1–4). Finally, we determined that deep ICH and labor ICH were causally related to PUD, and deep ICH was causally related to GERD.

## 4. Discussion

Previous studies have not found a clear causal relationship between stroke and gastrointestinal diseases. In our study, the relationship between stroke and its subtypes of gastrointestinal disorders was determined by the MR analysis. It is reported that obesity, smoking, anticoagulant, and other risk factors are often related to gastrointestinal diseases (39–41). The GWAS of GERD and PUD found genetic overlapping with the identified aforementioned hazardous factors (42, 43). We cannot rule out that SNP affects the outcome through other related variables. Therefore, we should try our best to reduce the bias caused by pleiotropy. To reduce pleiotropy, we look over the PhenoScanner and eliminate those pleiotropic genetic variants. Thus, we successfully removed SNPs that were highly correlated with possible confounders such as obesity, smoking, and anticoagulation therapy. In addition, we also conducted some sensitivity analyses, such as a leave-one-out analysis and a funnel plot, and other methods such as Cochran's *Q*-test and the MR-Egger intercept test to assess horizontal multiplicity.

Stroke is often associated with PUD. A retrospective review including 808 cases found that the incidence of gastrointestinal bleeding caused by PUD in patients with ICH was 26.7% (18). Moreover, the incidence of gastrointestinal bleeding was significantly higher in patients who often use stress ulcer prophylaxis (SUP) for stress ulcer prevention compared with patients not receiving SUP (18). Another observational study examined 177 patients with acute stroke by gastroscopy, of which 92 (52%) had gastric changes, 10 of which were acute ulcers (44). For patients with severe ICH, an observational study found that 28.0% of 715 patients with severe ICH developed stress-related gastrointestinal bleeding (SGIB) or stress ulcers during hospitalization (45). Regrettably, none of these observational studies had a large sample size. Our MR study suggested that stroke has a causal impact on PUD but only on deep ICH and lobar ICH and not ischemic stroke.

The pathogenesis of ICH complicated by peptic ulcers is still unclear and may be related to the damage to the thalamus and the subthalamus. To summarize various studies, the possible mechanisms are as follows: first, patients with acute ICH often experience intracranial hypertension and cerebral edema, which directly or indirectly causes damage to the brain stem, the hypothalamus, and other parts and finally affects their normal physiologic functions, leading to a dysfunction of the autonomic nervous system and gastric hyperchlorhydria. It lessens the blood flow of gastrointestinal mucosa and damages the gastric mucosal barrier, resulting in stress gastrointestinal ulcer peptic ulcers as well as peptic ulcer bleeding (46). According to previous studies, the development of stress ulcers in patients with ICH can be better predicted by the hematoma volume of ICH (47, 48). Mechanistically, larger hematomas in the case of cerebral hemorrhage are more likely to lead to increased intracranial pressure (45). As mentioned earlier, elevated intracranial pressure may cause strong sympathetic excitation and gastrointestinal vasoconstriction, causing a decrease in gastrointestinal blood flow, which subsequently leads to mucosal ischemia and increased gastric acid secretion. Second, post-stroke sepsis plays a very important role in the development of stress ulcers induced by severe ICH (45). Inflammatory cytokines are released in large amounts in the development of sepsis, thus exacerbating the ischemia of the gastrointestinal mucosa caused by intracerebral hemorrhage and driving the development of stress ulcers (49, 50). In an observational study, the incidence of gastrointestinal bleeding in patients with ischemic stroke was 7.8%, 74% of which were caused by peptic ulcers (51). Combined with our findings, it is clear that hemorrhagic strokes are more likely to develop peptic ulcers than ischemic strokes. The development of peptic ulcers in ischemic stroke may be associated with vagal hyperactivity, stress, and neuroendocrine dysregulation (51, 52). However, the trigger for gastrointestinal bleeding in most patients with ischemic stroke is not stress, and stress ulcers due to acute ischemic brain injury may be very rare (52, 53). One possible explanation for the aforementioned results is that compared to ischemic strokes, hemorrhagic strokes are a more devastating subtype of stroke (54). Hemorrhagic stroke may have a stronger effect on the brain–gut axis than ischemic stroke. The study finding that hemorrhagic stroke disrupts the gut microbiota more than ischemic stroke may prove this (55). The incidence of stress ulcer bleeding in patients with brain injury is closely related to the severity of the injury (56). In our MR study, the risk of lobar ICH is more associated with an increased risk of PUD compared to the risk of deep ICH. The size of the hematoma, sepsis, and prognosis have been reported to be the strongest predictors of gastrointestinal bleeding in patients with ICH in previous research (48). A Japanese observational study found a higher rate of poor prognosis in patients with lobar ICH than in those with non-lobar ICH (57). Even lobar ICH is associated with more severe cognitive impairment (58). It suggests clinical vigilance for PUD for hemorrhagic stroke.

Several studies have proposed an association between stroke and GERD. A population-based Taiwanese cohort study including 18,412 patients with stroke and 18,412 without stroke found that the risk of GERD in patients with stroke was 1.51 times higher than that in patients without stroke (95% CI, 1.40–1.67) (15). Moreover, they separated the stroke cohort into two subgroups: hemorrhagic stroke and ischemic stroke. Compared with the subjects without stroke, the HRs for GERD in the intracerebral hemorrhage and ischemic stroke cohorts were 1.45 and 1.52 (95% CI, 1.22–1.71 and 95% CI, 1.39–1.67) (15). Our MR study found that the higher risk of GERD is complicated by the risk of deep ICH, and there is a positive causal relationship between them. We reviewed the relevant literature to explain the mechanisms by which stroke leads to the development of GERD. For ischemic stroke, GERD may be induced by drugs used to treat IS, such as aspirin. One of the independent risk factors associated with the clinical symptoms of GERD is NSAIDs. The study also found an increased incidence of GERD in patients with stroke treated with antiplatelet therapy (15, 59). In addition, ischemic stroke may disrupt the neural regulation of oropharyngeal, esophageal, and gastrointestinal motility, resulting in an extensive impairment of oropharyngeal and gastrointestinal motility and a reduced tone of the lower esophageal sphincter (52). ICH has a similar effect on the vagus nerve, resulting in the malfunction of esophageal peristalsis, gastrointestinal motility, and the lower esophageal sphincter (48, 60). Parasympathetic dysfunction in patients with stroke may lead to impaired esophageal motility, the abnormal transmission of food, and the abnormal relaxation of the lower esophageal sphincter (15, 61). Hypertension is one of the most important risk factors for stroke, and treatment to lower blood pressure to prevent stroke, including the use of calcium channel blockers, often leads to lower esophageal sphincter (LES) pressure and eventually GERD (62, 63). A community study found that calcium channel blockers were independently associated with GERD symptoms as a risk factor (63). To explain why deep ICH is more prone to GERD than other subtypes of stroke, we looked through many studies. Deep ICH is often thought to be closely associated with hypertension, while lobar ICH is often thought to be caused by cerebral amyloid angiopathy (CAA) (64). Among the drugs used to treat hypertension, calcium channel blockers have the effect of lowering the pressure of the LES and impeding gastric emptying, thus inducing GERD (65). Therefore, compared to lobar ICH, deep ICH is more prone to GERD.

According to our MR results, intracerebral hemorrhage is more likely to cause gastrointestinal disease than ischemic stroke, and we are thinking about the reasons for this result. It is well-known that ischemic stroke and intracerebral hemorrhage do not occur by similar mechanisms, and their degree of criticality is different. ICH is the most severe subtype of stroke. Furthermore, the most devastating type of pathology among the subtypes of stroke is ICH (54). In general, ICH produces more severe strokes than cerebral infarct (66, 67). ICH typically manifests as elevated intracranial pressure, hematoma compression, and serious cerebral edema, which can cause many negative effects, such as neuroinflammation, mitochondrial dysfunction, and apoptosis, resulting in a sudden disruption of the blood–brain barrier (68). Contrary to ICH, the structural stability of brain cells and the blood–brain barrier is retained for a longer length of time following the beginning of symptoms in ischemic stroke (69). One possible explanation for our findings is that compared to ischemic stroke, ICH is more damaging to the brain–gut axis, causing a more severe dysbiosis in the gut microbiota, abnormal gastrointestinal motor function, and impaired gastrointestinal motility, which leads to gastrointestinal disorders. Compared to patients with ischemic stroke, patients with ICH have more severe gut microbiota destruction (70). ICH causes rapid damage to astrocytes and the blood–brain barrier in patients (69). Contemporary genetics considers stroke not as a disease but as a syndrome. Stroke is an acute manifestation of a range of chronic cerebrovascular diseases (71). Another possible explanation for our findings is that some subtypes of ischemic stroke present additional phenotypic dilemmas, such as the cardiogenic stroke subtype, whereas the phenotype of ICH is more uniform (71). Moreover, genetic factors are important in the pathogenesis of ICH (72). It is estimated that up to 44% of cases of ICH are heritable, and possessing an ICH first-degree relative increases the risk of developing the condition by a factor of six (73).

To explain the causal relationship between hemorrhagic stroke and several gastrointestinal diseases, we have found several possible mechanisms. Intracerebral smoke can affect the function of the autonomic nervous system. Through the enteric nervous system, the extrinsic and autonomic nervous systems can regulate the motor, sensory, and secretory functions of the gastrointestinal tract. ICH affects gastrointestinal function in this way, mainly with motor dysfunction (74). For example, strokes are often complicated by dysphagia, which may be due to cranial nerve involvement in the region of the vertebrobasilar artery (75). This is one of the possible causes of stroke complicating gastrointestinal motility disorder-related disease. Moreover, the change in gut microbes caused by intracerebral hemorrhage may be one of the causes of some gastrointestinal diseases (68). A prospective case–control study found that compared with the control group, the intestinal microbiota composition of both patients with ischemic stroke and patients with intracerebral hemorrhage changed (55). More specifically, compared with the control group, the abundance of invasive aerobic bacterial genera (*Enterococcus* species and *Escherichia/Shigella* species) in all patients with stroke increased, while obligate anaerobic genera decreased (55). The authors observed that the extent of gut microbiota destruction was positively associated with the severity of stroke. An intracerebral hemorrhage causes more severe disruption of the gut microbiota than an ischemic stroke (55). The autonomic nervous system abnormally releases norepinephrine to the intestine, which may change the intestinal microbiota (23). Another study found that the immune system of model mice is disturbed after intracerebral hemorrhage. Furthermore, the gut barrier function of model mice was impaired, and intestinal permeability increased (70). In addition, experimental studies also found that inflammatory cytokines were upregulated in the intestine, malondialdehyde (MDA) levels were elevated, the superoxide (SOD) dismutase activity was reduced, severe intestinal mucosal damage and plasma endotoxin levels were elevated 2 h after intracerebral hemorrhage in model mice, and intestinal propulsion was reduced 12 h later, and these symptoms persisted for 7 days after the appearance of the above symptoms (76). These suggest that intracerebral hemorrhage significantly increases inflammatory cytokine levels and myeloperoxidase activity, which, in turn, promotes an inflammatory response in the intestine, leading to gastrointestinal disorders associated with intestinal motility and barrier dysfunction. In contrast, elevated malondialdehyde levels and reduced superoxide dismutase also suggest that intracerebral hemorrhage induced excessive oxygen radical production in the intestine during ischemia-reperfusion. The pathological imbalance of the intestinal oxidative–antioxidant system may also be involved in the pathogenesis of gastrointestinal disorders after intracerebral hemorrhage (76). In a word, intracerebral hemorrhage may lead to impaired communication between the brain and intestinal axis, which may directly result in gastrointestinal motility dysfunction or intestinal flora disorders. Although there are many studies on how the brain–gut axis interacts, the exact mechanism has not been clarified.

Our MR study has some strengths. First, compared with one-sample MR, our research has a larger sample size and higher statistical efficiency. Second, our research overcame the shortcomings of traditional causal inference. Since the alleles followed the principle of random assignment, we obtained results independent of the confounding factors and reversed causal associations found in traditional epidemiological studies. Furthermore, there is Cochran's *Q*-test, the MR-Egger intercept test, and sensitivity analysis to test the pleiotropy of instrumental variables, which enhances the reliability of the results. At the same time, our analysis has some limitations. First of all, the estimates mentioned in our MR study cannot be directly compared with those of other observational studies. Second, we have selected only four common gastrointestinal disorders, and it is unknown whether a stroke has a causal effect on other gastrointestinal disorders. Third, the dataset on which our study is primarily based includes only individuals of European ancestry and thus may not be applicable to other humans, which would make our findings not generalizable. Finally, because of the limitation of the number of SNPs, the *p*-value limits were adjusted in our article.

Our MR study provides evidence for a causal relationship between deep ICH on PUD and GERD and a causal relationship between lobar ICH on PUD, and our results add to the gap in observational studies in this regard and warrant further research for the prevention of gastrointestinal disorders after deep ICH and lobar ICH.

## 5. Conclusion

Our research supports a possible causal link between stroke and its subtypes and gastrointestinal disorders. Early gastrointestinal disease risk assessment and prevention in hemorrhagic stroke is crucial and could aid in the introduction of tailored treatment as soon as possible.

## Data availability statement

The original contributions presented in the study are included in the article/Supplementary material, further inquiries can be directed to the corresponding author.

## Ethics statement

Ethical review and approval was not required for the study on human participants in accordance with the local legislation and institutional requirements. Written informed consent for participation was not required for this study in accordance with the national legislation and the institutional requirements.

## Author contributions

JS contributed to the methodology and wrote the manuscript. WC contributed to conceptualization and investigation. WY contributed to the funding, writing, reviewing, and editing. All authors contributed to the article and approved the submitted version.
