# Supplementary material for: Stroke and the risk of gastrointestinal disorders: A Mendelian randomization study
Source: Front Neurol. 2023 Feb 21;14:1131250. doi: 10.3389/fneur.2023.1131250 (PMC9989308; doi:10.3389/fneur.2023.1131250)

Supplementary Information

**Supplementary Figure 1.** Estimates of nominal significant results from ischemic stroke on gastrointestinal disorders. (A) Scatter plots from genetically predicted any ischemic stroke on GERD; (B) Scatter plots from genetically predicted small vessel stroke on GERD; (C) Leave-one-out plot from genetically predicted any ischemic stroke on GERD; (D) Leave-one-out plot from genetically predicted small vessel stroke on GERD; (E) Funnel plot from genetically predicted any ischemic stroke on GERD; (F) Funnel plot from genetically predicted small vessel stroke on GERD.


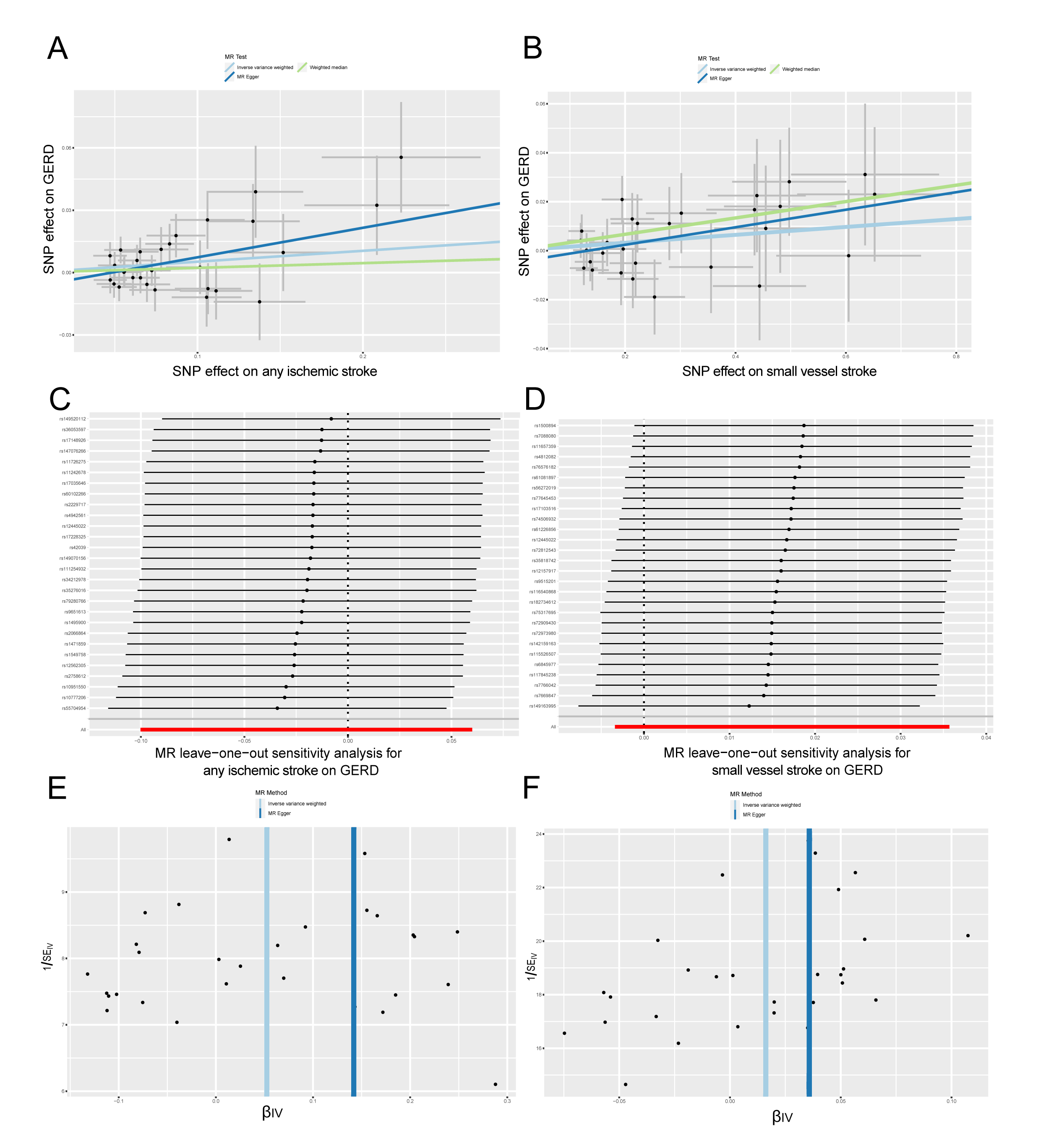


**Supplementary Figure 2.** Scatter plots of significant and nominal significant estimates from genetically predicted hemorrhagic stroke on gastrointestinal disorders. (A) All ICH on PUD; (B) All ICH on IBD; (C) Deep ICH on PUD; (D) Deep ICH on GERD; (E) Lobar ICH on PUD; (F) Lobar ICH on IBS.


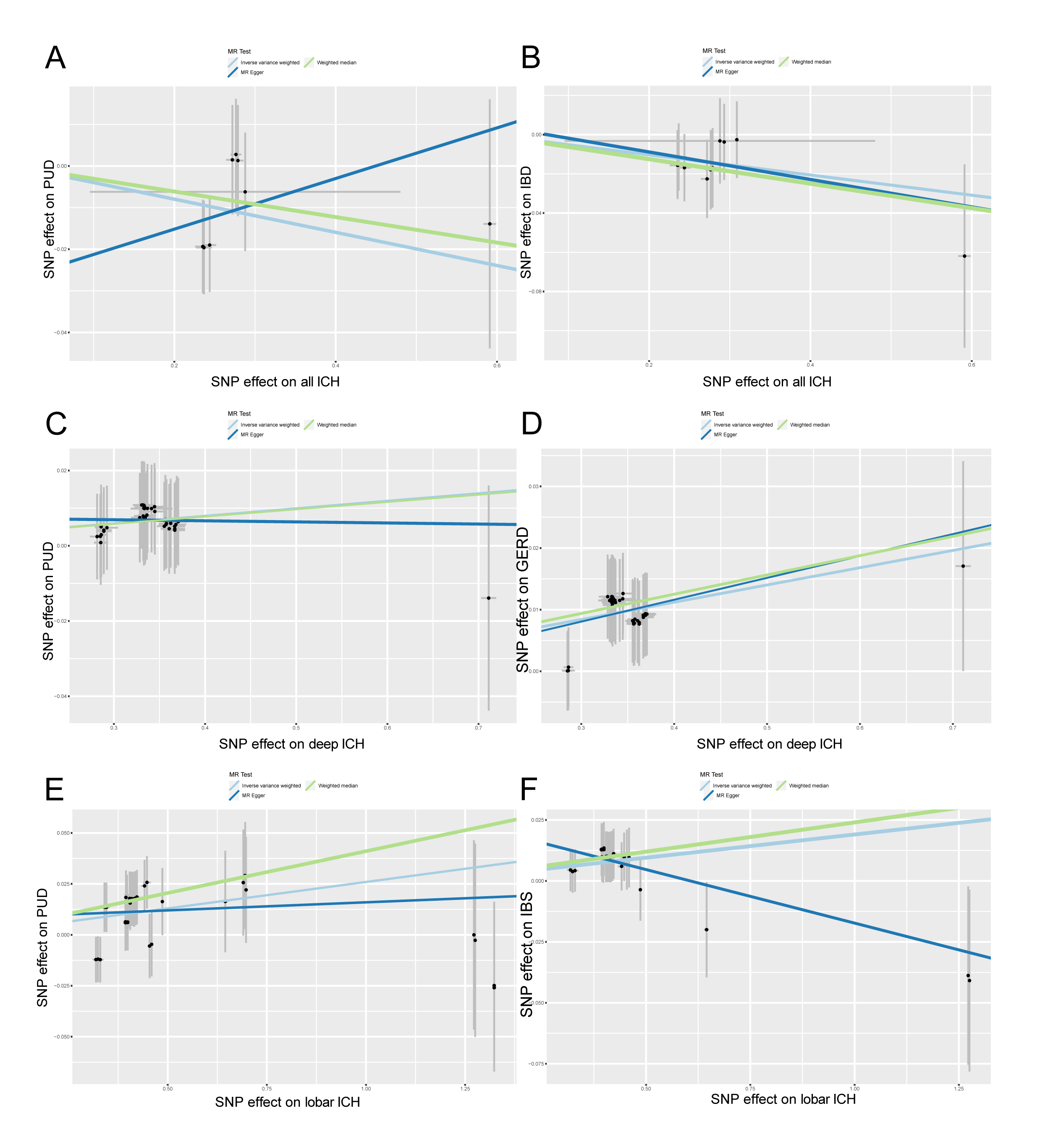


**Supplementary Figure 3.** Funnel plots of significant and nominal significant estimates from genetically predicted hemorrhagic stroke on gastrointestinal disorders. (A) All ICH on PUD; (B) All ICH on IBD; (C) Deep ICH on PUD; (D) Deep ICH on GERD; (E) Lobar ICH on PUD; (F) Lobar ICH on IBS.
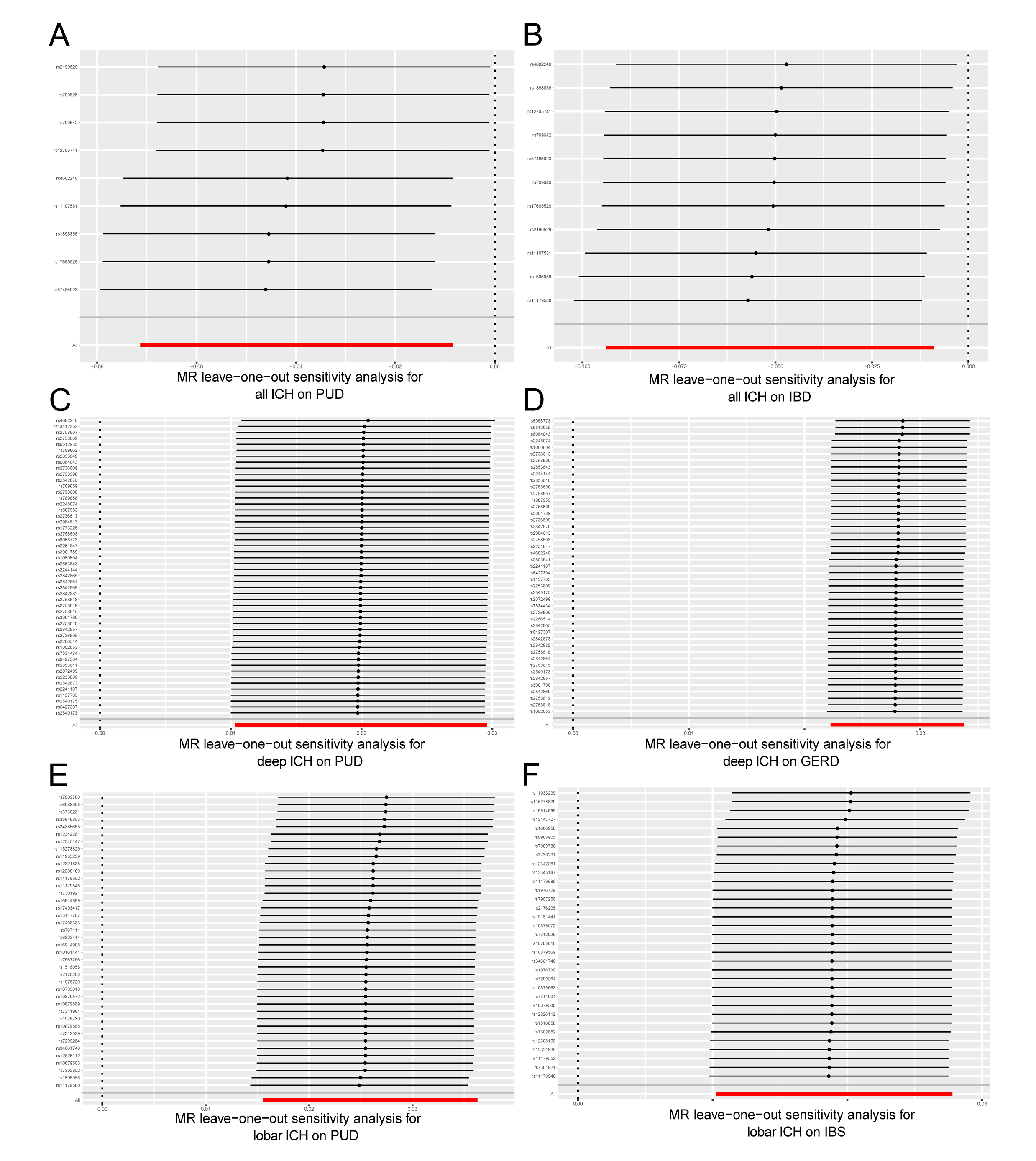


**Supplementary Figure 4.** Funnel plots of significant and nominal significant estimates from genetically predicted hemorrhagic stroke on gastrointestinal disorders. (A) All ICH on PUD; (B) All ICH on IBD; (C) Deep ICH on PUD; (D) Deep ICH on GERD; (E) Lobar ICH on PUD; (F) Lobar ICH on IBS.


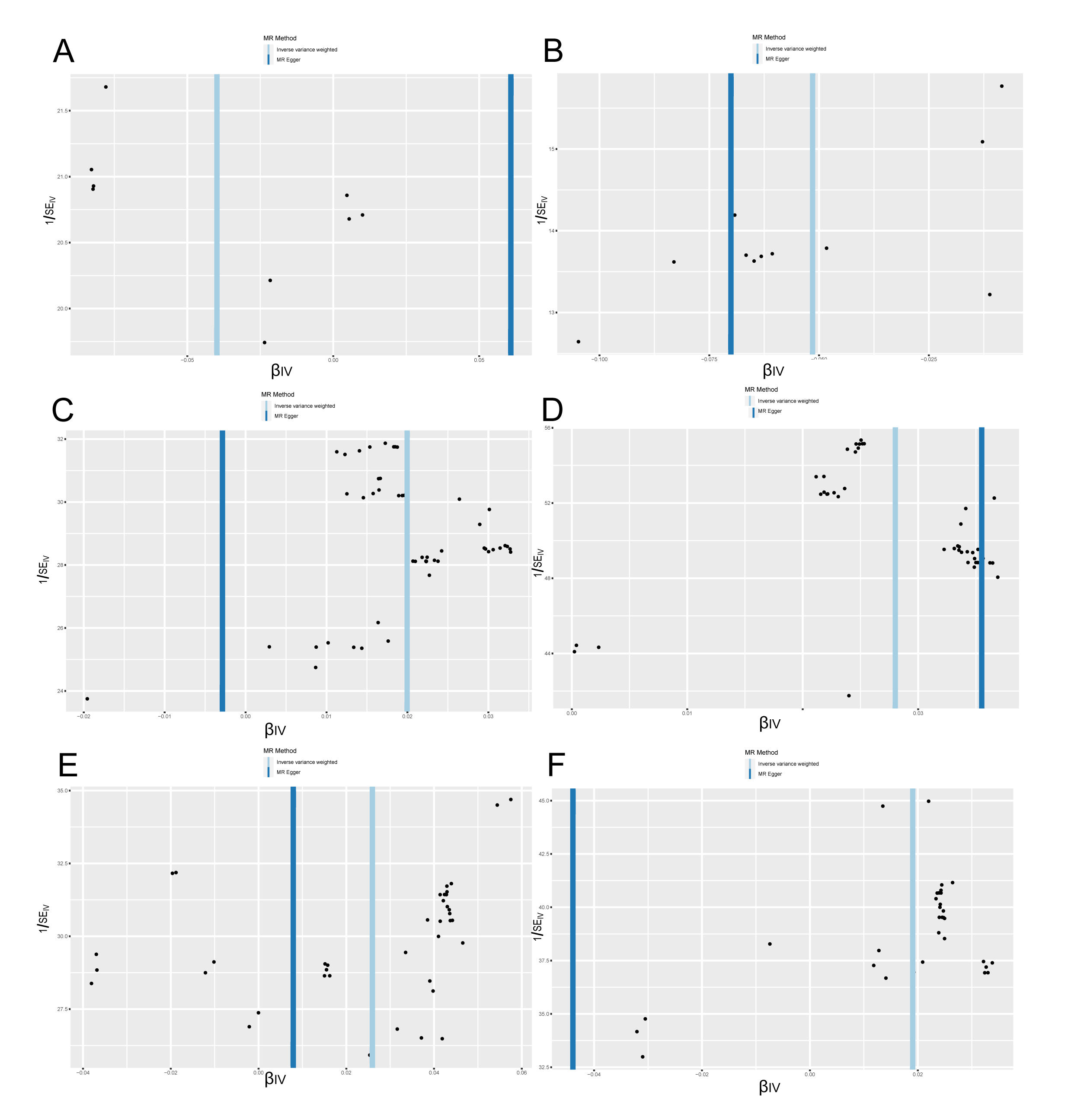

Supplement: Supplementary file 2 [file Table_2.DOCX]
